# Oxygen Sensing for Industrial Safety — Evolution and New Approaches

**DOI:** 10.3390/s141406084

**Published:** 2014-03-27

**Authors:** Martin Willett

**Affiliations:** City Technology Ltd., Walton Road, Portsmouth PO6 1SZ, UK; E-Mail: martin.willett@citytech.com; Tel.: +44-23-9228-8132; Fax: +44-23-9238-6611

**Keywords:** oxygen, sensing, electrochemical, lead, anode, pump

## Abstract

The requirement for the detection of oxygen in industrial safety applications has historically been met by electrochemical technologies based on the consumption of metal anodes. Products using this approach have been technically and commercially successful for more than three decades. However, a combination of new requirements is driving the development of alternative approaches offering fresh opportunities and challenges. This paper reviews some key aspects in the evolution of consumable anode products and highlights recent developments in alternative technologies aimed at meeting current and anticipated future needs in this important application.

## 1. Introduction

The ability to reliably measure oxygen concentration (or partial pressure) and provide warning of abnormal conditions is a critical requirement in industrial safety environments such as mines, oil production facilities and chemical plants. Hazards arise primarily because of the risk associated with asphyxiation on entering confined spaces where oxygen levels have become depleted by displacement, combustion, oxidation or other chemical processes. (Oxygen enhancement, for example in the vicinity of welding equipment, can also represent a serious risk). Oxygen measuring instruments containing electrochemical sensors based on consumable lead anodes have been available for over 30 years. Such devices provide early warning of unsafe conditions and are a key aid in attempts to reduce accidents [1–3].

Expansion in the use of gas detection instruments in industrial safety applications is driven by a combination of legislative, commercial and performance factors. Thus, the use of lead is now seen as undesirable given the increasing restrictions on the use of the metal resulting from its health and environmental impacts [4]. In addition, end users are constantly searching for methods to reduce the costs associated with meeting their gas detection needs. Oxygen sensor manufacturers must therefore deliver:

- Lead-free sensing technologies;
- Price reductions in real terms;
- Increased longevity and reliability;
- Improved performance across increasingly wide environmental ranges and
- Reduced calibration and test requirements.

In this paper, technical developments which have helped to meet these demands are discussed. Specific emphasis is placed on methods which are well aligned with the constraints dictated by use in small, portable single- and multi-gas detection instruments. A number of lead-free methods will also be reviewed and future development routes considered.

## 2. Consumable-Anode Sensors—Success through Iterative Improvement

Amperometric lead-based electrochemical sensors operate on principles which broadly resemble those governing a primary metal-air battery. The relevant core technology elements have been discussed elsewhere [5]. The key reactions are electrochemical reduction of oxygen at the sensing electrode:

$${\text{O}}_{2}+{2\text{H}}_{2}\text{O}+{4\text{e}}^{-}\to {4\text{OH}}^{-}$$

and reaction of lead with hydroxyl ions at the counter electrode:

$$2\text{Pb}+{4\text{OH}}^{-}\to 2\text{PbO}+{2\text{H}}_{2}\text{O}+{4\text{e}}^{-}$$

**Figure 1.**
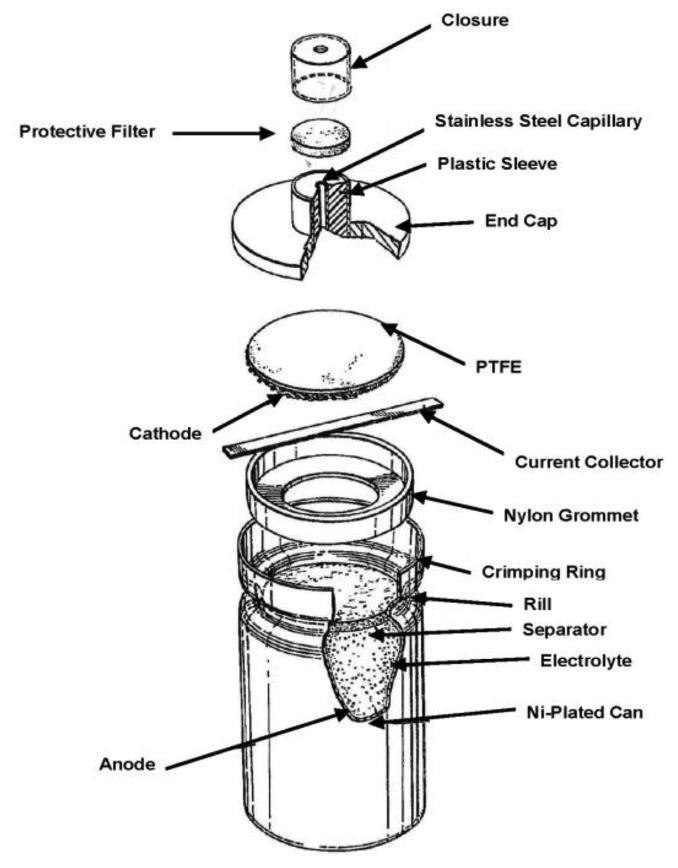
Early version of lead-based electrochemical sensor (modified from [8]).

Although electrochemical technology had long been proposed as a basis for gas detection [6], key advances in the design of gas diffusion electrodes [7] and the introduction of gas phase diffusion barriers in the late 1970s [8] were key enablers for the first truly practical designs. Early commercial products such as those marketed by City Technology (London, UK) in 1977 [9] (Figure 1) offered a compelling combination of attractive features for instrument designers and end users which none of the competing technologies available at the time could surpass. In contrast, solid electrolyte devices based on ZrO_2_ such as those used in automotive applications operated at high temperatures (>600 °C), leading to unacceptable power consumption, whilst paramagnetic sensors were insufficiently robust and too costly for widespread portable field use.

Although the initial driver for development was to provide personal protection in industrial environments [10], it was rapidly recognised that reliable low cost oxygen sensors could offer significant opportunities in monitoring of emissions from small combustion plants and so assist in improving fuel efficiency. Medical applications in anaesthesia and neonatal care have also benefited from the same technologies. These and the many other applications for electrochemical oxygen sensors are not the primary focus here, but they continue to present additional challenges which have driven oxygen sensor technology forward.

These early lead-based electrochemical sensor products offered:

- Low voltage and current supply requirements, compatible with implementation in small portable instruments (simplest approaches can be self-powered);
- Low power consumption, allowing continuous instrument operation throughout a typical working shift (8 h) without needing to change or recharge the battery;
- Relatively low cost;
- Orientation insensitivity;
- Rejection of carbon dioxide interference;
- Operation across an acceptable environmental range;
- Good linearity and the ability to design for operation in different oxygen ranges;
- Pressure coefficients and transients which could be managed/compensated;
- Relatively low temperature coefficient (T^1/2^ dependence);
- Options for concentration or partial pressure designs with appropriate gas phase diffusion barriers (capillary or Knudsen regimes).

Since such early lead-based products became available, there has naturally been a gradual rise in customer expectations, driven by the need to offer performance meeting new or more stringent requirements. This has required technological developments in a number of key areas.

### 2.1. Sensor Electrolyte Modelling

**Figure 2.**
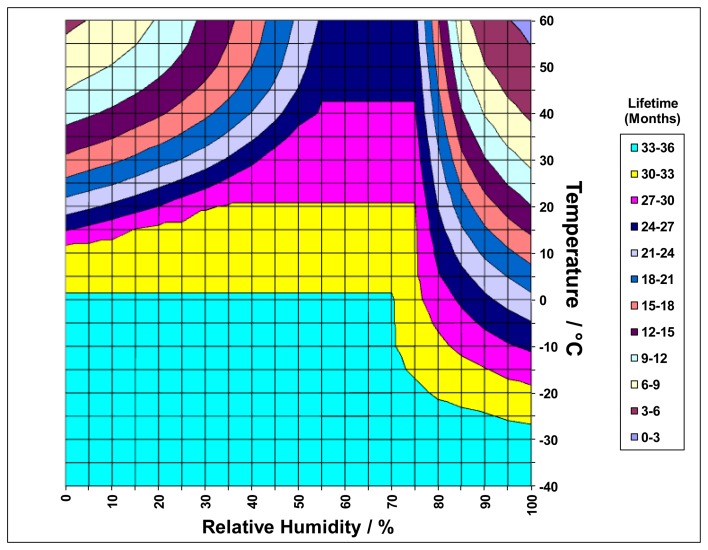
Example of lead-based sensor model outputs. Lifetime predictions for continuous operation at selected temperatures and relative humidities (modified from [11]).

As user industries operate in more extreme environments, sensors have been required to function for longer periods under harsher conditions. One of the main limitations is the loss and take-up of water from the atmosphere by aqueous electrolytes. For example, it is known that key operational parameters such as the response time and sensitivity degrade as a function of water loss, whereas water take-up can result in sensor failure once all the free volume within the cell has been filled. In order to understand this process more fully and provide accurate predictive tools, detailed water management models have been developed to relate sensor failure modes to the environmental exposure of the device. Using iterative methods based on knowledge of:

- electrolyte vapour pressure;
- temperature and relative humidity of the environment;
- cell diffusion barrier properties and
- component material properties,

a comprehensive model has been developed which can predict the safe useable life of different designs [11]. An example of the output is shown in Figure 2, providing a valuable tool for advising users on optimum product selection and routine maintenance planning.

Further evolution of such models is currently underway, employing finite element methods to model the location and concentration of electrolyte throughout the sensor stack. In conjunction with data gathered on failure modes of particular designs, this approach allows novel design solutions to be evaluated in a virtual environment without the need to rely solely on empirical prototype data as was traditionally the case. In addition to improving the reliability and speed of developments, this information will facilitate intelligent operational systems able to provide automatic warning of maintenance requirements and modify sensor outputs to extend operating life in extreme operating conditions. Such approaches provide tools which can equally be applied to the electrolytes commonly employed in conjunction with alternative anode materials (Section 2.4) and oxygen pump sensors (Section 3).

### 2.2. Anode Utilisation and Pressure Equalisation

In any coulombic-limited system, there is a conflict between the magnitude of the normal output in air and the lifetime which can be extractwed from an anode having fixed size. This applies to lead-free systems and to traditional designs. It has long been recognised that a key challenge in anode selection is to achieve a good balance between:

(a) the fundamental energy density offered by the metal (taking due account of reaction stoichiometry, molecular weight, density *etc.*);
(b) the available reaction surface area;
(c) passivation which can occur as a result of the reactions on which operation depends and
(d) the ability to handle the material efficiently in a manufacturing environment.

One particular issue with lead is the increase in volume which occurs on oxidation due to the lower densities of the oxides. This can reduce the effectiveness of operation toward end of life if not factored in to the anode design. Powder anodes can give higher surface areas but do not provide optimum accessibility of the electrolyte throughout large volumes. For this reason it has become common to use lead in a stranded form which can be easily and cheaply compressed into the required shape whilst offering extremely high utilisation (>95%).

In lead systems using a single gas access immediately above the sensing electrode, the design intent is that the lower parts of the sensor should operate in essentially anaerobic conditions apart from relatively small quantities of gas dissolved in the electrolyte bulk. However, such designs present challenges in terms of venting pressure differentials which build up between the internal cell volume and the external environment when the sensor is exposed to temperature transients. Undesirable output “glitches” can occur when gas flows through the capillary to equalise the expansion or contraction of gas within the sensor, a situation which is inconsistent with the diffusion principle under which the design is intended to operate.

This can be addressed by providing a vent [12], but two further issues must then be considered. Firstly, additional parasitic consumption may occur by virtue of oxygen accessing the cell without passing over the sensing electrode and which therefore represents a non-mass transport controlled contribution to the output signal. This must be taken into account when calculating the capacity of the anode and hence the sensor lifetime. Secondly, loss of contact can occur between the lead strands and the external connections in the vicinity of the vent due to aerobic reactions. Flow soldering of the anode base has been used to protect regions where there is greatest risk of reduced connectivity [13]. As a result, practical designs eliminating “glitching” behaviour have been realised.

### 2.3. Capillary Barrier and Control of Bulk Flow

Although porous gas phase diffusion barriers had been demonstrated using a variety of membranes with appropriate pore sizes, it was quickly recognised that single capillaries were simpler to implement in practical designs. A key challenge for early manufacturers was therefore the development of a method to reliably fabricate small capillary barriers offering the required combination of properties. The barrier must limit the rate of oxygen ingress so that the sensing electrode is not kinetically limited. This naturally reduces the sensor output signal but increases the useable life from any given anode.

**Figure 3.**
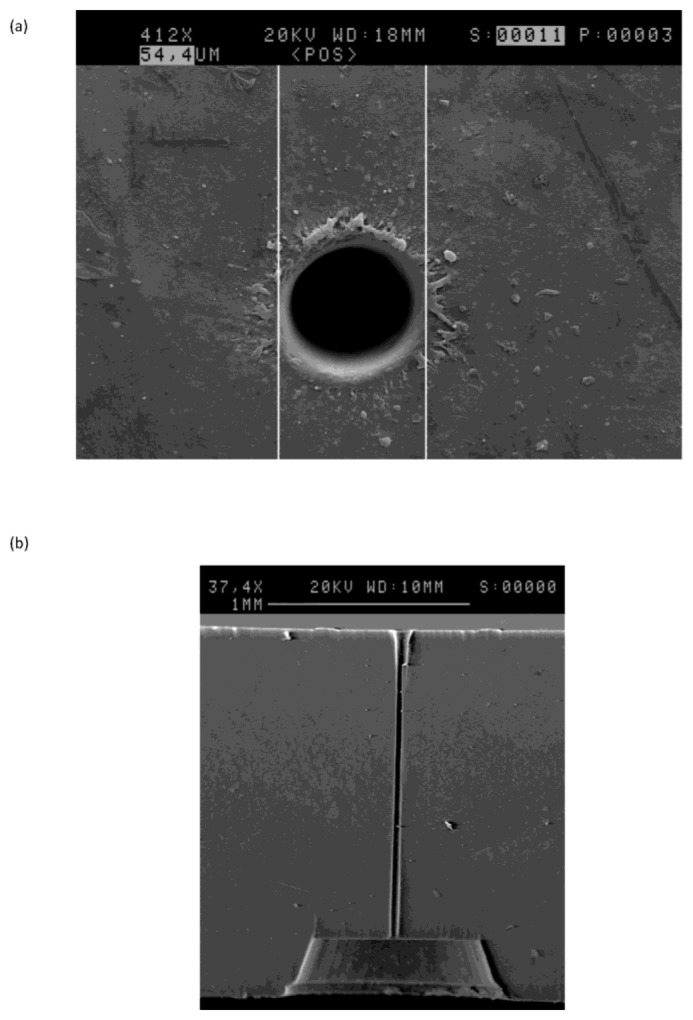
Example entry aperture (**a**) and cross section (**b**) of laser drilled capillary diffusion barriers for oxygen sensors.

**Figure 4.**
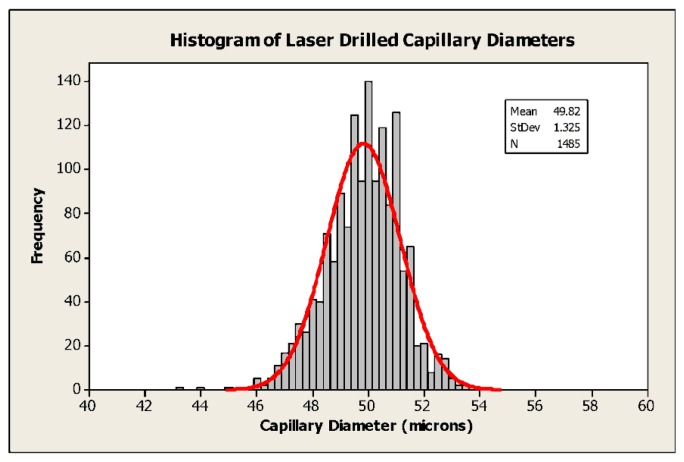
Sensor capillary diameters—typical reproducibility of development samples.

Many early sensors employed capillaries fabricated using steel hypodermic tubing or, later, by drawing wire through a molten soldered cap. Typical dimensions were 200–300 μm diameter by 5 mm in length [8], so that a C-size sensor (50 mm in length by 26.2 mm in diameter) was only reliably capable of a few months' life. Although a marked improvement over the state of the art at the time, this was quickly recognised as a limitation. In addition, the poor reproducibility of the capillary dimensions resulted in sensors having a relatively wide range of outputs which then needed to be individually calibrated and tuned by the user. Improvements in mechanical drilling techniques and the introduction of plastic moulded housings (in place of the original metal battery cans) allowed holes of ∼ 130 μm diameter to be produced in ∼3 mm long capillaries and contributed to further improvements in life. For the past few years, it has been commonplace to employ laser drilling, for example with a frequency doubled Nd:YAG laser at 532 nm (typical power output 5 W). The examples shown in Figure 3 are about 50 μm in diameter and 1.5mm in length, and can be manufactured with high reproducibility, as shown in Figure 4. Each evolution of capillary fabrication improved sensor life by reducing current levels in normal atmospheric environments containing 20.9% oxygen, allowing the use of smaller anodes. Sensors having life of >3 years can now be manufactured in an envelope ∼50% smaller than that of products introduced in the early 1980s using the same fundamental chemistry, whilst simultaneously offering numerous other performance improvements.

Although outside the scope of this discussion, sensors based on the use of thin, non-porous membrane barriers relying on solid state diffusion of oxygen (giving an output dependent on partial pressure) offer a different set of manufacturing and performance opportunities and challenges [5,14]. They are widely used in other application fields (such as emissions monitoring and medical measurements), but industrial safety users invariably prefer the concentration output of capillary barrier sensors, and relevant performance standards are usually expressed in terms of percentage oxygen by volume [15].

The performance of a capillary diffusion limited sensor relies on the assumption that gas reaching the sensing electrode is fully consumed and that the output signal is controlled by mass transport. However, when a capillary oxygen sensor is subjected to a sudden pressure increase or decrease, gas is forced through the barrier, resulting in a bulk flow. This disrupts the diffusion limitation, resulting in an enhanced (or reduced) flux of gas into the sensor and hence a current transient on the measured signal. Although such effects quickly decay to zero once diffusion conditions re-establish, the effect can generate false alarms, which are unacceptable. As capillary dimensions become smaller and the sensitivity of the cell per % O_2_ is reduced, the relative impact of the pressure equalising flow becomes greater. Therefore, the drive to smaller capillaries for lifetime improvements has required the simultaneous development of methods to control these effects.

One highly effective solution to this problem is to include a bulk flow membrane below the capillary. Under static conditions, this has minimal effect on the sensor current as it presents a much lower diffusion resistance (due to its high porosity) than the capillary. However, it has high resistance to bulk flow (by virtue of its small average pore diameter) and so damps the transient to an acceptable degree.

### 2.4. Alternative Anode Materials

Although lead was recognised from the outset as offering an attractive combination of properties suitable for commercially viable designs, a range of other metals can in principle be used as anodes. Cadmium, bismuth and copper were identified as potential options by early designers [8]. Some of these materials offer significantly greater energy densities toward the oxygen reaction than lead (and hence potentially longer life from an anode of fixed size). From the current perspective it may seem odd that a greater variety of successful alternative designs have not appeared in the 3 decades since lead based sensors became popular. To some extent, this can be explained by the conservative nature of the legislation-driven health and safety industry, where it is common for early products meeting a clearly identified need to become the method of choice. (Some of the candidates are also subject to restrictions on use due to their toxicity.) The barrier for competitive technologies to displace existing approaches which have been adopted in standard operational practice should not be underestimated. There are also major technical challenges in creating a true drop-in replacement for the standard 2-terminal self-powered lead sensor which can be used in existing instrumentation without requiring extensive redesign and recertification.

The requirement for lead-free solutions has increased as a result of pending legislation restricting the use of the metal [4]. There is growing interest in the use of oxygen pump designs (as discussed in Section 3), but alternative consumable anodes have also received considerable attention, and the options can be divided into 2 categories. Group I includes metals where the normal operating potential of the anode permits water reduction and hydrogen evolution in aqueous electrolytes, leading to parasitic consumption of both the electrolyte and anode. Group II metals sit above the hydrogen evolution line of the respective Pourbaix diagrams and are generally free from such effects in normal operating conditions. Although novel nonaqueous electrolytes offering freedom from such concerns are slowly becoming available, they are still only used in a minority of consumable anode cells. The discussion here will focus on solutions compatible with the more common aqueous systems.

#### 2.4.1. Group I

The first group of metals including tin, aluminium and zinc have some attractive properties as the basis of consumable anode sensors in alkaline [16], neutral [17] and acidic [18] systems. Indeed, zinc offers the highest energy density of all readily available alternatives. However, these metals suffer from self-corrosion (leading to hydrogen evolution) or passivation of the anode surface in aqueous systems which compromises their performance. In order to suppress these effects, two routes have been proposed. The first is to move away from simple two electrode self-powered operation and operate the cell in a three electrode potentiostatic circuit. By using an appropriate reference electrode, the potential of the anode can be biased to a point where hydrogen evolution can be reduced to acceptable levels [19]. A drawback of this approach is a lack of compatibility with existing designs. This is not such a major issue in multigas instruments where the use of biased toxic sensors is commonplace—although the design and certification changes required are still a barrier to entry for new products. However, in small, zero-maintenance single gas devices, the simple unpowered consumable anode system offers significant advantages. In addition, the recent appearance of reliable oxygen pumps (which also require bias) provides a non-consumable long life option for those adopting potentiostatic control of oxygen sensors. As a result, biased consumable systems have found little acceptance.

An alternative approach is to incorporate additives in the electrolyte and/or anode to suppress the water reduction processes and hydrogen evolution of these metals (e.g., for tin [16]). Investigation of a wide range of additives and coatings to inhibit zinc corrosion in conventional alkaline electrolytes such as aqueous potassium acetate has also been undertaken [20]. A number of potential candidates have been identified, but none of these are able to provide a complete solution without requiring other significant compromises in performance.

#### 2.4.2. Group II

A second group of anode candidates are provided by metals in which corrosion in aqueous environments is not thermodynamically favourable, for example Bi, Cu or Sb. The theoretical basis for this assertion has been discussed elsewhere [21] and predicted lifetimes have been estimated [22], indicating that a relatively low mass of the metal is required compared with that needed in a lead cell having equivalent life. In the absence of premature failure modes, continuous operation in a standard two electrode cell for 5 years at a current of 100 μA requires approximately 6.5 g Sb, 10.5 g Cu, 11.4 g Bi or 16.9 g Pb. This offers the prospect of extended operating periods for the same sized sensor compared with current devices but without the drawbacks of corrosion associated with the options discussed in Section 2.4.1.

Practical demonstrations of these approaches have been realised using analogues of conventional lead based sensors built with alternative anodes and 4M KOH electrolyte [23]. This presents some challenges since Cu is the only one of the chosen candidates readily available in a high surface area wire form. Bi and Sb are relatively brittle and anodes were therefore processed from solid blocks or fabricated from packed beads.

It is well known that consumable anode sensors primarily fail due to either: (a) a fall in output as the accessible anode is completely consumed (or the surface becomes unavailable due to inhibition processes) or (b) increasing response time as the ability of the anode to match the demands of the sensing electrode becomes limited. Setting pass/fail criteria for each parameter allowed the behaviour of the prototypes in accelerated aging tests under 100% O_2_ to be compared. This confirmed that Sb offered the longest life once due account was taken of the anode surface areas. It had also been predicted that metal surface passivation would be a likely failure mode, which XPS studies of the antimony oxides appeared to confirm through the presence of Sb_2_O_3_ layers.

Using solid, perforated and two close packed bead designs (approx 2 and 1 mm diameter), antimony anodes were produced with similar overall dimensions (allowing use of the same sensor hardware) but having surface areas of about 500, 900, 2,200 and 4,400 mm^2^, respectively. The lifetime under different oxygen concentrations confirmed that the integrated charge passed by the sensors before failure was approximately proportional to the available surface area (passivation reactions halted anode consumption well before the available mass had been consumed). This is in agreement with the proposal that lower current densities at the anode surface should help prevent the onset of passivation caused by the build-up of oxidation products. Thus, sensors having anodes of the same mass but higher surface area would have longer life when used at the same capillary limited current. The smallest beads gave an estimated life of ∼5.7 years continuous operation at 100 μA, and it is anticipated that further improvements could be obtained with suitable processing of the anode material.

### 2.5. Summary

There have been major technological and manufacturing advances in consumable anode technologies over the past 30 years and they continue to play a dominant role in industrial safety markets. There are a number of alternative approaches to the consumable anode concept which offer the prospect of longer lifetime from lead-free versions of such sensors, although to date these have achieved comparatively little market penetration. This may alter as legislative issues become more pressing. However, competition from new technologies is expected to significantly alter the landscape in the coming years and the following sections discuss some of the key approaches.

## 3. Oxygen Pump Technology

Electrochemical oxygen pump sensors have been available for many years—automotive oxygen emissions sensors based on ZrO_2_ were in use in the early 1980s [24]. In common with lead-based sensors, they too have evolved continuously to deliver improved performance, life and reliability at reduced cost. However, the overwhelming majority of such devices rely on the use of solid ionic electrolytes which require high operating temperatures (>600 °C). The associated power consumption renders them unattractive in the vast majority of industrial safety applications.

**Figure 5.**
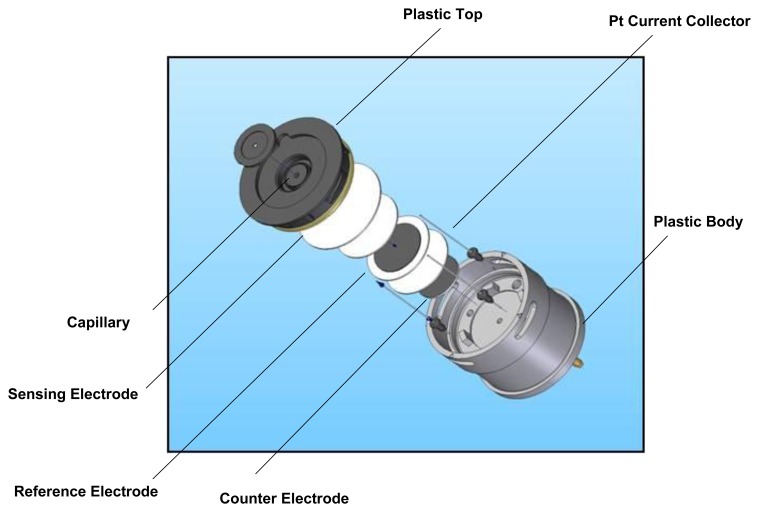
Early design for liquid electrolyte fuel-cell oxygen pump.

It has long been recognised that the core technology of fuel-cell liquid electrolyte electrochemical sensors of the type widely used for toxic gas measurement [5] lends itself to the detection of oxygen. They generally employ acid electrolytes and gas diffusion structures for the sensing, counter and reference electrodes (Figure 5) linked to a conventional potentiostatic control circuit. Gas phase diffusion barriers are employed, based on the assumption of complete consumption of the target gas reaching the sensing electrode. This invariably requires the use of a biased sensing electrode, which is seen as a drawback. However, this is counterbalanced by the removal of the coulombic capacity limitation, since pump sensors in principle have no consumable components. Their practical life is set by material and manufacturing issues rather than by fundamental technological limitations. In some applications, warranties of up to 10 years are offered on electrochemical CO toxic gas sensors utilising the same core technology as oxygen pumps [25]. The impending restrictions on lead use and the perceived weaknesses of alternative anodes have provided fresh impetus to the development of oxygen pumps and the resolution of their unique challenges [26–29].

### 3.1. Counter Venting and Oxygen Distribution Modelling

In a pump, oxygen is reduced at the sensing electrode and evolved at the counter electrode:

$$\begin{array}{c} \text{Sensing}—\;{\text{O}}_{2}+{4\text{H}}^{+}+{4\text{e}}^{-}\to {2\text{H}}_{2}\text{O}\\ \text{Counter}—\;{2\text{H}}_{2}\text{O}\to {4\text{H}}^{+}+{4\text{e}}^{-}+{\text{O}}_{2}↑ \end{array}$$

A primary concern is therefore that oxygen evolved at the counter electrode should not feed back to the sensing electrode and produce erroneous readings. One way of achieving this is to ensure that a pathway having low gas diffusion resistance is provided from the counter to an external vent in the sensor housing which is distant from the main inlet capillary. The intention is that oxygen preferably exits the cell in the gas phase rather than being dissolved in the electrolyte. Although diffusion rates are much slower in the liquid phase (typically by a factor of 10^4^), it can be difficult to control the precise location of the oxygenated electrolyte as hydration levels vary with environmental conditions and so ensure full screening of the sensing electrode. If the vent is positioned at the opposite end of the stack to the primary gas access, then the cell can be partitioned to eliminate direct gas paths between the sensing and counter electrodes. Ionic conductivity can be maintained via a porous material designed to remain saturated with electrolyte.

Additional precautions are required to avoid possible problems arising from sensing—counter crosstalk when the sensing and counter electrodes are in close proximity within the cell (for example printed onto a common gas diffusion substrate). In such cases it is often found helpful to introduce a long pathlength labyrinth connection between the two, so that ionic connectivity can be maintained but reducing the risk of oxygen migration in the liquid phase. A further option is to introduce an additional scavenging or screening electrode to consume any oxygen migrating toward the sensing electrode from the counter, but this adds cost and complexity.

**Figure 6.**
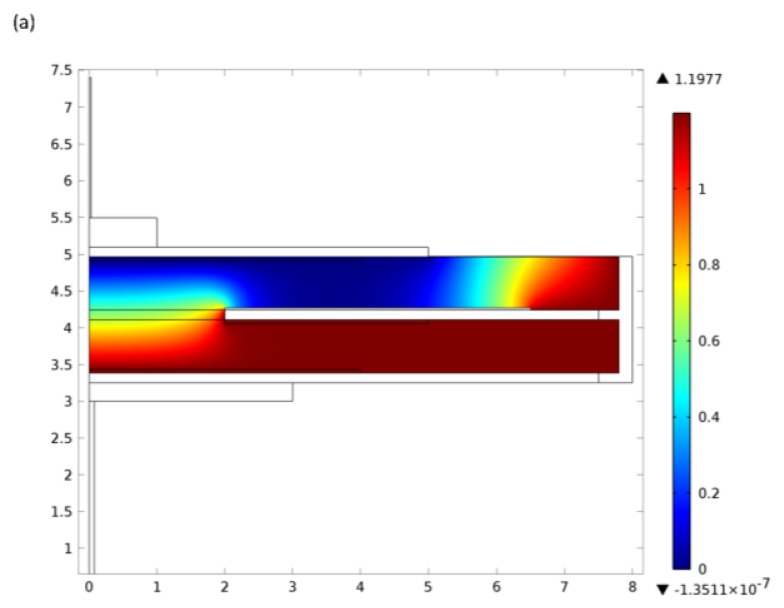

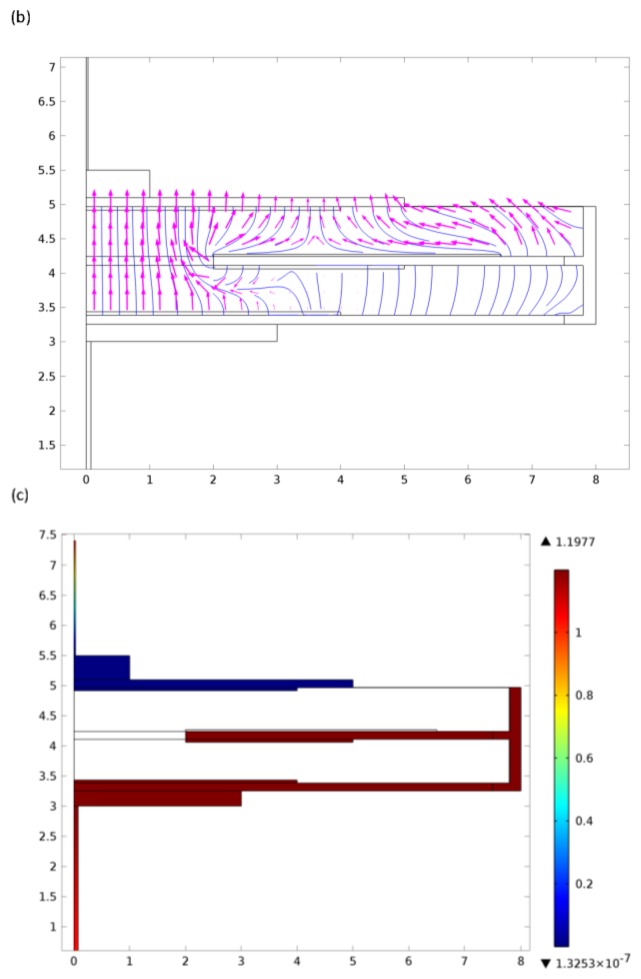
Example of electrochemical oxygen pump model outputs. (**a**) Dissolved oxygen distribution; (**b**) Dissolved oxygen flux; (**c**) Gas phase oxygen distribution.

Knowledge of oxygen concentrations within the sensor (both in the gas and liquid phases) is critical. To obtain a better understanding of the behaviour as a function of external conditions, and so optimise electrode and stack geometries, finite element models have been developed. These allow the prediction of many key performance parameters for a wide range of designs as a function of time, electrolyte properties and environmental conditions. A typical example is shown in Figure 6.

### 3.2. Power Consumption and Start up

User resistance to the requirement for oxygen pumps to run at bias is not primarily driven by a lack of acceptance of biased sensors *per se*, since these have been successfully employed for many years in toxic gas detection. However, there are key differences when comparing a pump with primary cells which continue to operate (and consume their anodes) as long as the load resistor remains connected.

All biased electrochemical sensors demonstrate gradual start-up after being off load for significant periods. This results from the fact that the electrodes drift to their natural rest potentials which must then be re-established by consuming the species which would not normally be present at the operating bias. Additionally, and more importantly, oxygen is normally present in significant concentrations in the working or storage atmosphere experienced by the pump. This is the opposite scenario to that which usually exists for toxic gases. Therefore, when a pump is switched off and the sensing electrode reactions stop due to the bias no longer being applied, oxygen accumulates within the sensor and will eventually equilibrate at 20.9% with the external atmosphere given enough time. Not only does this oxygen fill the gas phase voids within the cell, it also dissolves in the electrolyte. When bias is reapplied, there is a large reservoir of oxygen which must be consumed before normal diffusion limited operation (driven by consumption of all oxygen reaching the sensing electrode) can be re-established. This results in significant and unacceptable delays, especially in comparison with most other sensors in common use. The expectation for start-up times in portable instruments is typically <30 s to provide a useable signal.

As a consequence, it is highly desirable that power to bias the oxygen pump is maintained even when the instrument is switched off. Oxygen pumps have a small power consumption—the potentiostat drain for a 100 μA output sensor is typically <0.3 mW which is negligible compared with that of catalytic sensors normally present in multigas instruments. Therefore, the impact of this requirement upon the run time which can be achieved from the instrument battery is negligible in these cases.

The same is not true of low cost single gas portable instruments for oxygen detection. In such devices, consumable anode sensors are packaged with non-rechargeable batteries capable of powering the system display and alarms for the full operational life of the unit without recharging [30]. In these units, even a relatively low pump power consumption will have a significant effect on useable life. This is currently an active area for research—a number of novel solutions to resolve the start-up problems have been proposed and are currently being evaluated.

Although the start-up behaviour can represent a problem, the same effects can also be used as diagnostic techniques to determine whether sensor components are performing within expected limits [31]. In general terms, the method involves reducing or halting the gas reaction at the sensing electrode, allowing unreacted gas to accumulate, and then resuming the reaction, whilst analysing the rate of decay of the transient. The diffusive and kinetic behaviour of the sensing electrode, the behaviour of the bulk flow membrane and the performance of the primary diffusion barrier can all be probed by employing variants of the technique. To achieve useful results, it is only necessary to reduce the sensing electrode activity for a very short period or to apply small bias deviations. Such methods are therefore compatible with the demands of field operation.

### 3.3. Water Management and Novel Electrolytes

Given that oxygen pumps currently rely on a similar range of aqueous electrolytes to those used in consumable systems, water management is again of significant concern. The sensing, counter and reference electrodes all require an adequate supply of electrolyte and the design of reservoirs and wicking systems to contain and transport electrolyte is critical. In toxic gas fuel cells, which share many of the same requirements, the relatively large capillary plays a dominant role in water exchange with the atmosphere, but as the capillary diameter is reduced, a number of other effects become important.

Thus, in oxygen pumps relying on liquid barriers to prevent counter-sensing electrode crosstalk, maintaining hydration of the barrier is particularly important in seeking to prevent “crossover”. This is the situation which arises when the barrier becomes partially dried, opening up direct gas paths with relatively low diffusion resistance. Such behaviour is characterised by a significant increase in output due to oxygen evolved from the counter reaching the sensing electrode.

**Figure 7.**
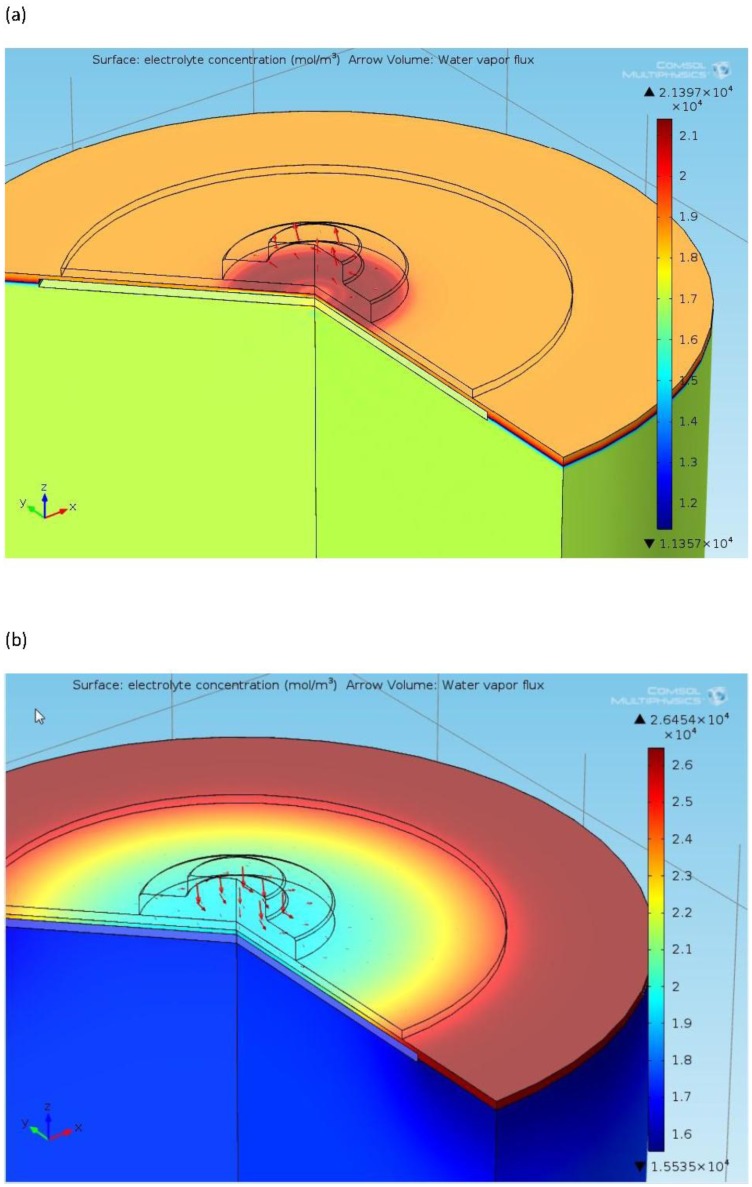
Examples of electrochemical sensor water management model outputs. Spatial electrolyte distributions showing water vapor fluxes at (**a**) 20% RH; 50 °C and (**b**) 80% RH, 10 °C.

**Figure 8.**
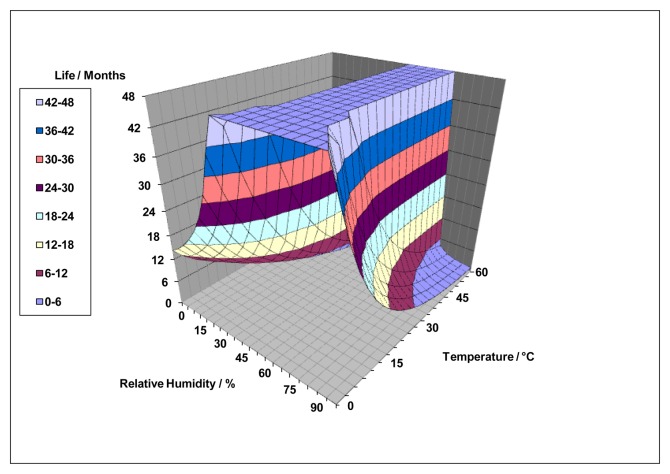
Example of electrochemical oxygen pump model outputs. Lifetime predictions for continuous operation at selected temperatures and relative humidities.

Finite element water management models exploring the movement and properties of the electrolyte have been used to characterise the behaviour of electrolyte in structures of this type and to define material properties required for optimum performance (Figure 7). By combining these with oxygen distribution models, comprehensive insight into sensor behaviour may be exploited to predict lifetimes (Figure 8) and improve designs.

In addition to improved utilisation of existing electrolytes, there is considerable interest in the use of novel electrolytes which can alleviate some of the drawbacks of aqueous systems. Ionic liquid electrolytes [32] and solid polymers [33] are increasingly exploited in toxic gas fuel cells. Although there are some key differences between the requirements for different measurands, it is anticipated that future oxygen pumps will also utilise these improved materials to deliver enhanced performance.

### 3.4. Summary

The development of electrochemical oxygen pumps has accelerated in response to concerns over the use of lead and the perceived shortcomings of alternative anodes in consumable systems. The drawbacks associated with the requirement to bias the sensors and concerns over start-up are gradually being overcome and the prospect of highly reliable, long life sensors is a compelling argument for change. Most sensor manufacturers now expect such pumps to become a major (and possibly the dominant) oxygen sensing technology in industrial safety applications in coming years.

## 4. Other Technologies

### 4.1. Luminescence Quenching

Materials which exhibit luminescence quenched by the presence of oxygen have been known for many years, and have long been recognised as offering a basis for the fabrication of selective oxygen sensors [34]. In recent years, a number of reviews have described various material systems which can be exploited, including polycyclic aromatic hydrocarbons, metalloporphyrins and polypyridyl complexes [35,36]. Ruthenium, platinum and palladium compounds are amongst the most frequently employed in these applications.

The development of miniaturised, low power consumption sensors has been greatly enhanced by the improved availability in recent years of suitable optical sources (typically blue LEDs) and photodetectors. Given that decay times for the luminescent transitions employed in these systems tend to be of the order of microseconds, relatively fast processing electronics are also required for successful implementation. Related approaches are also well known in the field of optochemical sensing using optical fibres to relay light to and from the dye [37,38]. However, fibre methods currently offer little practical advantage in the development of small portable personal instrumentation for industrial safety users and will not be discussed in detail.

To date, the most common applications in gas detection have been in food storage, where the ability to interrogate a dye sample through transparent packaging allows non-invasive analysis of controlled atmospheres. However, the environment experienced by the luminescent material in such cases is relatively benign when compared with that in most industrial plants. Thus, a focus for development of the technology has been in understanding and improving the robustness and stability of the dye/host system performance over long periods, exposure to common pollutants and other potential interferences. The temperature sensitivity of many of the favoured material systems can represent a particular problem, and a number of approaches have been proposed to address this issue [38,39].

The use of appropriately matched polymer hosts can facilitate the development of more robust and stable luminescent films for industrial oxygen sensing, although more long term field experience in harsh environments is still required. Ongoing improvements in optical sources and detectors (allied to fast electronics) now allow integration into industry-standard packages [40], potentially addressing user concerns regarding cost and power consumption. Indeed, low duty cycle pulsed operation of LED sources can be achieved at powers similar to those required by electrochemical oxygen pumps whilst eliminating start-up transients. Conversion of the partial-pressure dependent luminescence output to a more desirable concentration response can be readily achieved using miniature integrated barometers. These developments suggest that luminescence quenching may offer a viable alternative to other powered oxygen detection approaches in future.

### 4.2. Direct Optical Absorption

Oxygen exhibits weak optical absorption in the near IR at ∼760 nm and a number of manufacturers have developed absorption based systems capable of delivering sensitivity adequate for industrial safety purposes [41–43]. However, these use narrow band laser sources in order to provide good signal to noise ratios, which places them well outside the cost/complexity envelope acceptable to portable instrument manufacturers. LEDs are readily available at these wavelengths but their emission spectra are too broad to act as the basis of low cost systems with the required performance. (This is in contrast to the situation which exists for hydrocarbons or CO_2_ which have strong mid-IR absorptions. In these cases, filament bulb or LED sources are employed in small NDIR sensors [44–47] having power consumption compatible with portable instrumentation.)

Performance improvements and cost reductions in laser sources at wavelengths useful for oxygen detection are certainly anticipated. However, it seems likely that direct optical absorption methods will remain confined to high value niche applications in the industrial safety market for the foreseeable future.

## 5. Conclusions

Since the appearance of practical, low cost, lead-based electrochemical oxygen sensors in industrial safety applications over 30 years ago, customers and legislators have naturally increased the demands placed upon these products. Sensor manufacturers have responded by constantly innovating and improving their designs to deliver performance enhancements, longer life, greater reliability and cost reductions in real terms. The technology has been one of the key enablers in the availability of small, compact instruments for use in industrial safety markets. It is conservatively estimated that since 1980, approximately 50 million sensors of this type have been sold world-wide and they continue to dominate this key industrial application. However, legislative changes and challenging new demands are now combining to drive the development of alternative approaches.

Whilst the comparatively simple, zero power solution offered by consumable lead anode sensors remains attractive in terms of instrument design and operation, impending legislation means that alternative anode materials must be employed within a few years. Although several replacement options have been demonstrated, none currently offer a compelling technical route to the delivery of oxygen sensors having the greatly increased lifetime and reduced size required by users. It is expected that the market share of consumable anode systems will decline as older equipment based on their use is gradually replaced.

The new market demands are most likely to be met by oxygen pumps based on fuel cell technology. Many of the relevant design features are well proven from long term field experience of toxic electrochemical sensors, and further size reductions are feasible via improved electrolyte management and novel materials. The circuit redesigns and power consumption demands of the approach are unlikely to outweigh the other advantages, as illustrated by growing market penetration. The take-up of electrochemical oxygen pump sensors is expected to accelerate in coming years.

Other technologies cannot currently match the combination of performance, power and cost achievable with oxygen pumps. Luminescent sensors are the most likely group to offer a viable alternative, but their deployment is currently hindered by a lack of data to demonstrate longevity and robustness across a wide range of challenging industrial environments. Until there is much greater field experience of the approach, users in safety-critical applications are likely to prefer the proven capabilities of electrochemical systems.
